# Hybrid Virtual Commissioning of a Robotic Manipulator with Machine Vision Using a Single Controller

**DOI:** 10.3390/s22041621

**Published:** 2022-02-18

**Authors:** Marek Noga, Martin Juhás, Martin Gulan

**Affiliations:** Institute of Automation, Measurement and Applied Informatics, Faculty of Mechanical Engineering, Slovak University of Technology in Bratislava, 812 31 Bratislava, Slovakia; martin.juhas@stuba.sk (M.J.); martin.gulan@stuba.sk (M.G.)

**Keywords:** hybrid virtual commissioning, digital twin, robotics, simulation, machine vision, PLC

## Abstract

Digital twin (DT) is an emerging key technology that enables sophisticated interaction between physical objects and their virtual replicas, with applications in almost all engineering fields. Although it has recently gained significant attraction in both industry and academia, so far it has no unanimously adopted and established definition. One may therefore come across many definitions of what DT is and how to create it. DT can be designed for an existing process and help us to improve it. Another possible approach is to create the DT for a brand new device. In this case, it can reveal how the system would behave in given conditions or when controlled. One of purposes of a DT is to support the commissioning of devices. So far, recognized and used techniques to make the commissioning more effective are virtual commissioning and hybrid commissioning. In this article, we present a concept of hybrid virtual commissioning. This concept aims to point out the possibility to use real devices already at the stage of virtual commissioning. It is introduced in a practical case study of a robotic manipulator with machine vision controlled with a programmable logic controller in a pick-and-place application. This study presents the benefits that stem from the proposed approach and also details when it is convenient to use it.

## 1. Introduction

In recent years, automation has undeniably gained a significant role in many engineering fields. The effort to speed up and simplify the processes as much as possible necessarily implies the requirement to make the time needed for design, development and commissioning a device as short as possible. It is also similar in the case of designing new machines or even entire production processes. There is a number of different approaches and procedures on how to achieve the desired result. The arrival of the COVID-19 pandemic, which markedly affected the market and the entire economy, has exposed even more the advantage of using digital tools to reveal or propose possible solutions to the impacts of various events [1]. To a great extent, they use the concept of digital twin.

### 1.1. Digital Twin

In the literature, one can find many definitions of a DT; however, there is none that is recognized both in academia and across industry sectors [2]. At the same time, there are many approaches to the creation of a DT, see, e.g., [3,4,5,6]. In short, according to Schluse et al. [7], DT can be understood as a representation of a real object or subobject with its data, functions and means to communicate in a digital environment. Qin et al. [6] describe the DT in a similar way and add that DT can also be used for process optimization, monitoring, diagnostics and predictions based on artificial intelligence (AI), machine learning and software analysis. DTs are hence used for simulation and assessment of system or process behavior, based on which it is possible to take appropriate actions. Note also that, within the Industry 4.0 concept, AI is very often used together with DT [8]. The use of DTs for manufacturing systems can be aimed at the support of technical condition analysis for the purpose of improvement and predictive planning of maintenance activities, management and optimization of performance of devices during their lifecycle [9].

Before starting to create a DT, it is necessary to ask questions, in particular, whether it makes sense and what benefits it would bring. Given our requirements on the model, is it even necessary to create a DT? Would the investment into DT return or would it be lossy? It may also happen that creation of DT will result in a model that is too complex, which tends to be the case when striving for a DT model identical to the device [10].

Every simulation requires power and time to perform computations. When designing a DT, it is therefore of utmost importance to consider which parts of the device or process are meaningful to simulate and which in turn would be useless and only lead to an increase in computational effort. There is of course an effort to approximate the real system as good as possible. It is therefore necessary to know what precision to achieve already at the design stage.

All these factors significantly affect the required resources—computational, time, financial and last but not least, human.

Although there is no uniform definition of a digital twin, an international standard ISO 23247 is currently published. This standard defines a framework to support the creation of digital twins of observable manufacturing elements including personnel, equipment, materials, manufacturing processes, facilities, environment, products and supporting documents. It consists of four parts [11]:

- ISO 23247-1: General principles and requirements for developing digital twins in manufacturing;
- ISO 23247-2: Reference architecture with functional views;
- ISO 23247-3: List of basic information attributes for the observable manufacturing elements;
- ISO 23247-4: Technical requirements for information exchange between entities within the reference architecture.

**Figure 1:**
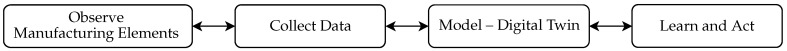
Principle of creating a digital twin according to ISO 23247.

The main principle of creating a DT according to ISO 23247 is illustrated in Figure 1.

In the first step, the standard assumes observing the device or process for which a model is to be created. Based on this, it shall be determined which signals and data shall be obtained from the process and further integrated into the DT model. Next, based on this model we shall decide how to improve the process itself, i.e., what actions shall be taken in order to improve its behavior or performance.

We refer the interested reader to [12] for a demonstration of various use case scenarios for digital twin implementation based on ISO 23247.

Another possible approach is described in [13], according to which a DT shall consist of the following four parts:

- Real space;
- Virtual space;
- The data link from real space to virtual space;
- In addition, an information link from virtual space to real space and virtual sub-spaces.

Note that the digital representation of a physical entity can be also built from multiple parts. This approach allows to create a detailed physics-based simulation model for the purpose of process analysis [13].

### 1.2. Virtual Commissioning

Digital twin is often associated with the concept of virtual commissioning (VC). It denotes an approach when virtual prototypes of devices are used to validate functionality of the program as well as the mechanics [14]. The employment of virtual commissioning brings many other advantages that include, e.g., avoiding the risk of damaging the device during commissioning while ensuring safety of the personnel.

**Figure 2:**
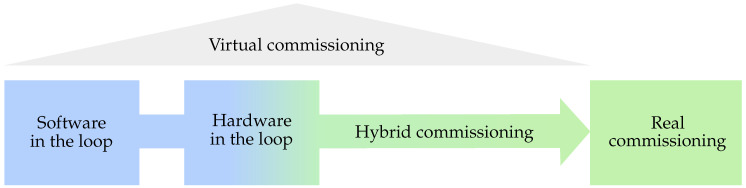
Variants of virtual commissioning. Reproduced with permission from authors of [15].

Virtual commissioning typically builds on two main approaches: software in the loop (SIL) and hardware in the loop (HIL). These are followed by hybrid commissioning (HC), which eventually leads to real commissioning, as illustrated in Figure 2.

Within the SIL approach, in addition to the simulated process, control hardware is emulated as well. It is conveniently used in both testing and design phase, since not a single piece of hardware is required, and programming of software is accelerated [16]. It is thus a purely off-line analysis where all hardware components are simulated.

Within the HIL approach, real control hardware is connected to the real-time simulation of the virtual devices. This enables to test even complex automation tasks with the control system that is to be utilized in real commissioning. The HIL simulation can be used at different levels of production [14].

Hybrid commissioning proposed in [17] represents an incremental procedure, which starts as HIL and stepwise replaces virtual devices by real devices and thus leading to real commissioning. In [17], the authors introduced the HC approach by means of an example of a PROFIBUS driven production plant where they compare and combine the signal values of real and simulated components.

### 1.3. Motivation

Let us now recall the ISO 23247 standard and its implementation presented in [12] and demonstrated by means of three use case scenarios. The first two, “Machine Health Digital Twin” and “Scheduling and Routing Digital Twin”, run above a real-world process or machine and focus more on their optimization or Product Lifecycle Management (PLM). It is however the third use case scenario, “Virtual Commissioning Digital Twin”, which is the most interesting from the perspective of this study. It differs by not having a real-time connection to the device. Specifically, it describes the use of VC for a CNC machine with a programmable logic controller (PLC) which is not physically constructed yet, but it is possible to perform its VC for various scenarios using a DT. The use case example assumes the HIL approach for this purpose. It also presents possibilities of using simulation environments to acquire appropriate signals for testing or verification and validation (V&V). The authors note that after performing all the tests, it is possible to deploy such a program on the real device.

Verification and validation of simulation models represent an integral part of the development process. The model, ergo the DT, is created for a specified purpose. Even before its design, it is therefore necessary to formulate questions that the system will be asked or tasked to solve and that the model has to be able to answer.

**Figure 3:**
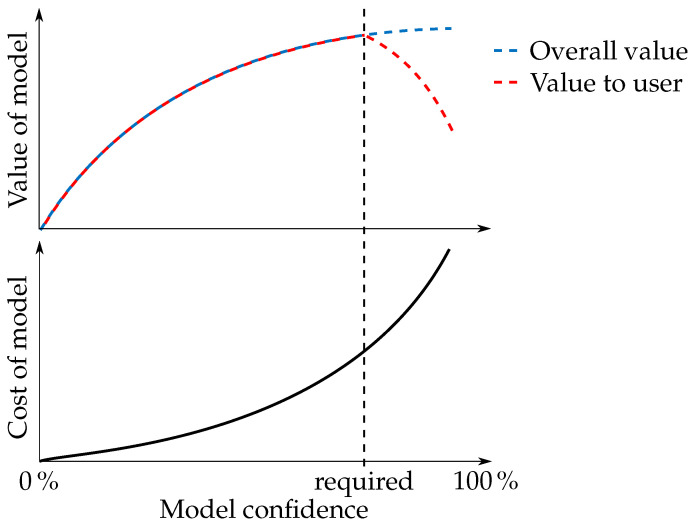
Illustration of relationship between value/cost of the model and its confidence.

In [18], the author discusses different approaches to V&V of simulation models. He also points out the fact that, despite that a model can be valid for various uses, it does not guarantee its validity for use in all applications in a given field. Figure 3 illustrates the relationship between value and cost of a model and its confidence. It implies that, beyond a required model confidence, its value to the user starts to decrease rapidly, while the time required to create the model has a dependence similar to the overall value of the model. Note that the cost of the model tends to be quite significant, especially when a very high model confidence is required. It is given by a sum of all incurred costs, from development to V&V.

A particular form of V&V is essentially the VC approach, which enables to shorten the time needed for programming and to resolve various software errors—and in conjunction with a suitable simulation environment, also construction defects. The connection of VC and DT offers an effective perspective on the product lifecycle. VC, however, also entails some drawbacks. In particular, it is to a great extent affected by accuracy of the model. Low accuracy can cause grave problems during commissioning and thus increase both its cost and required time. Hence, although VC can significantly accelerate the development process, it may happen that its cost will be too high, especially in cases of increasingly complex devices.

How would it thus be possible to lower the cost while maintaining the advantages of VC? One of the options is to save on the number of used software, and thus on the number of licenses as well as necessary programmers. Another option is to employ our proposed concept of hybrid virtual commissioning (HVC).

## 2. Hybrid Virtual Commissioning

HVC represents an approach combining VC with features of HC. Its main idea is to reduce the cost of simulation by using the available equipment, the cost of which can be lower than the license fees, and to simulate only the parts that are less affordable or currently unavailable. Use of this approach makes sense if the utilized devices or parts are so complex that it is more convenient to directly use them than to simulate them. Such a solution then requires even less time for commissioning since it allows for a more accurate testing by using DT, which further increases the likelihood of detecting defects.

In comparison with VC, HVC does not require a purely virtual model but combines it with available devices which do not need to be modeled or simulated. In comparison with HC (as described in [19]), HVC does not assume a successive transition from VC to HC but a direct use of components already in the VC phase. Similar to VC, HVC enables to employ both the SIL and the HIL approaches. Likewise, it can be followed by application of HVC, which will also require less time.

**Figure 4:**
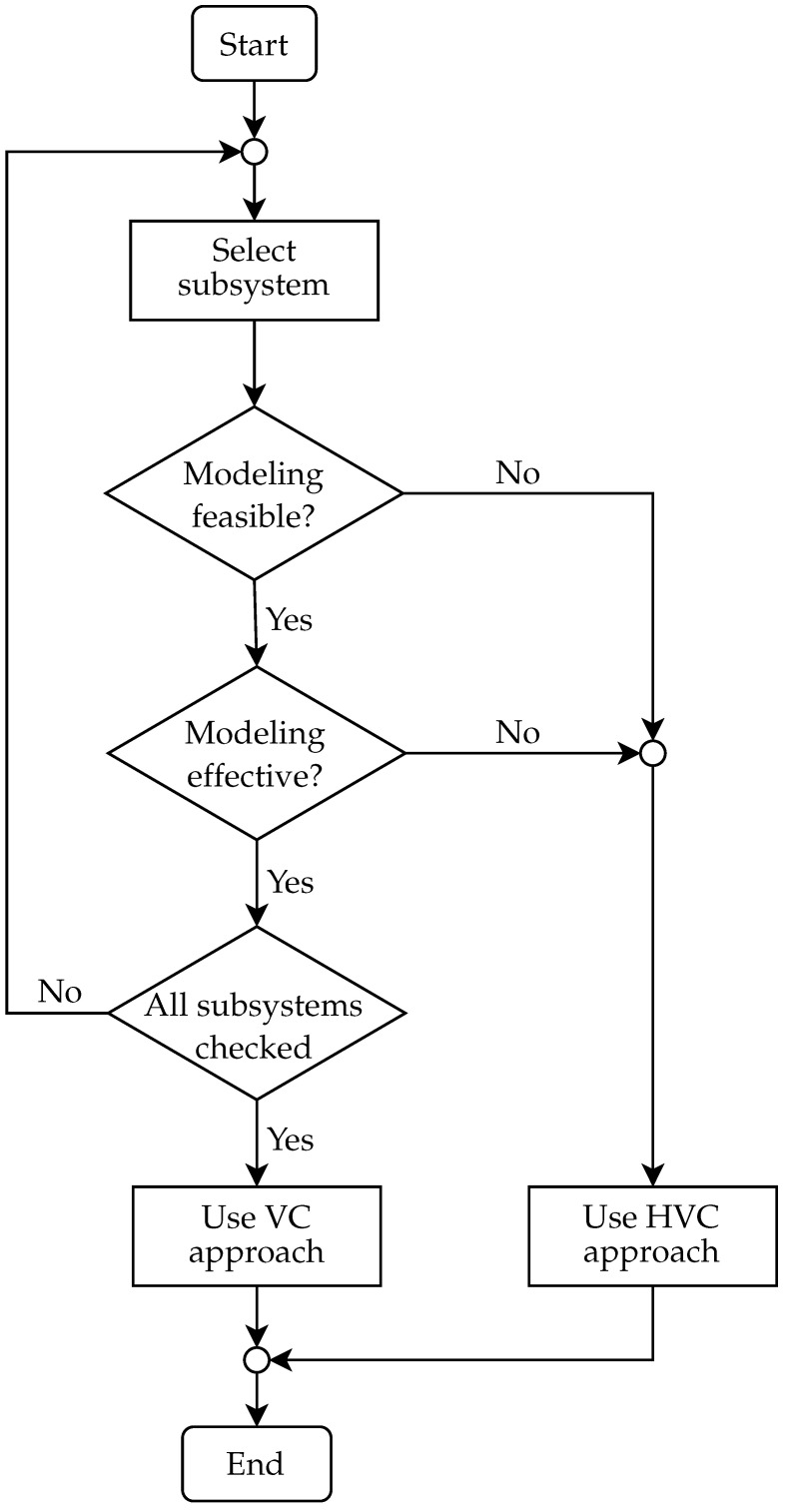
Flowchart of the proposed commissioning approach including HVC.

Figure 4 shows a flowchart for deciding which commissioning approach to choose. The feasibility of modeling can be understood as a software limitation, i.e., absence of license and other. At this step, it is necessary to consider the cost factor. Unless software limitations are an issue, efficiency of modeling needs to be taken into account as well.

Feasibility involves the overall complexity of modeling and therefore poses a question as to what amount of resources is required to model of a given element. The higher the complexity, the more demanding the feasibility—which, in most cases, implies more time needed for modeling and increase in costs, either of labor or hardware/software equipment used for modeling. During the entire decision-making process, one has to keep in mind that a model for DT is concerned; therefore, its repeatable use for future purposes needs to be considered as well. If it turns out that it is efficient to use at least one physical device (except for the control element, which would imply only a change from SIL to HIL simulation) in a subsystem, we no longer deal with VC but HVC.

It may seem that this concept does not fulfill the requirements for DT. If we, however, recall the definition of a DT, e.g., “A digital twin is a virtual representation of a physical object or system across its lifecycle, using real-time data to enable understanding, learning and reasoning” [20], it holds that also by using this approach it is possible to gather from DT data about behavior of the system during its commissioning but also at a later stage of lifecycle.

Our concept will be presented in more detail in a case study using a delta robot with machine vision and PLC control. Delta robots belong to a class of robotic manipulators with fast dynamics and are therefore suitable for tasks where their speed can be benefited from [21]. The most common in practice are pick-and-place applications where the delta robots replace the mostly monotonous manual labor. A pick-and-place task typically consists of handling of objects transported on a conveyor belt, while the necessary position information necessary for their gripping is acquired by a machine vision system. Note that particular systems need to be properly interconnected, while required resources will differ depending on used technologies.

Our device, situated in the authors’ “Learning factory for Industry 4.0” [22], combines the proposed solutions to reduce costs by employing HVC as well as by using less software environments. Validation of our model is performed by means of animation of DT. According to [23], it is possible to use the tracing method for the purpose of V&V. The author describes this method as a dynamical representation of the simulated system. In order to use the tracing, it is however assumed that the creators are well familiar with the real-world system the model corresponds to. In such a case, they shall be able to reveal errors in the control program.

**Figure 5:**
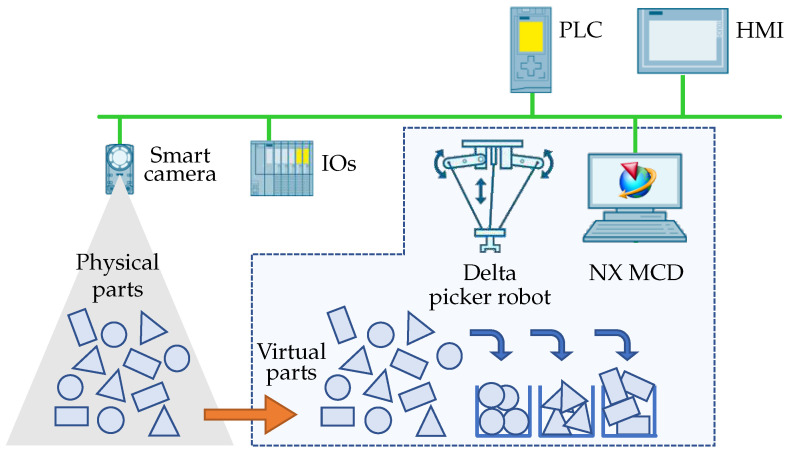
Conceptual design of the delta robot workplace used in our case study.

Figure 5 illustrates the proposed conceptual design of our device that will be subject to hybrid virtual commissioning. The reference task represents a modern use of robot manipulator in a pick-and-place application in combination with machine vision. The objective of the robot is to pick randomly positioned objects and to sort them into containers according to their type. The system for object recognition that determines the type and position of parts is realized by a smart camera. A fully commissioned solution requires physical availability of all of its elements—mechanics of the manipulator, gripper, work space with the parts and sort containers, servo drives, control system, as well as the machine vision system. This raises the price of possible mechanical adaptations, optimization of robot arms and way of gripping the parts, as well as hinders parallel commissioning of software during development and production work on the mechanics. It is therefore logical to employ the virtual commissioning approaches. Modeling of the camera system intended for this device would be hardly feasible and, given its availability, also ineffective. The HVC concept seems optimal in this application since it enables to mirror the physical assembly of parts scanned by the real camera into the virtual world of the digital twin, which leads to a full realization of the environment for the pick-and-place application, i.e., robot with gripper, containers and sorted parts. As it can be observed, the purpose of the device is to scan the position of objects in real time and to subsequently convert them into digital space, where virtual commissioning of the delta robot will take place. Its task is to sort the objects based on their position data. To achieve an effective and reliable control, we propose to create a digital twin of the delta manipulator.

### 2.1. Used Components

The computer providing necessary power for running the simulation environment, the programming environment and the OPC UA server features a 3.1 GHz i7-8705G CPU and 32 GB of RAM.

For the purpose of controlling the delta manipulator, we chose a Siemens PLC from the S7-1500TF series, which provides sufficient computing power for control of multiple technology objects (TOs). Each version of the technology series offers a limited number of technology sources. These can be combined in various ways, which allows to simultaneously control a larger number of even different TOs. The PLC enables to also create direct or inverse kinematics calculations of motion or to use some of the predesigned kinematic TOs. One of them is delta manipulator, and calculations for its TO are based on the inverse kinematics problem. This PLC can also process a safety program for the automation tasks and allows to create an OPC UA server. In our case, OPC UA server SIMATIC Net is used for communication between the controller and the real-time simulation environment. In Section 2.4, we will discuss how the choice of server affects the real-time simulation itself.

Programming of PLC is performed by means of the TIA Portal platform. Specifically, the project is programmed in version 15.1. This work is greatly simplified by a good availability of libraries and examples that can be used in one’s own program. This environment also enables to program a Human–Machine Interface (HMI) which allows effective operation and control of the device and displays information about its state.

For the input-output (IO) signals, a distributed IO system SIMATIC ET 200SP is utilized. It is connected via Profinet network to the PLC where IO signals are processed.

Information about the position of physical parts is acquired from a smart camera, namely SIMATIC MV540 by Siemens. The camera is capable of recognizing digital codes as well as objects. It uses the Profinet protocol for data communication. Its configuration via web interface, specifically Web Based Management (WBM), and integration into TIA Portal facilitate the programming.

In order to create a DT of the manipulator, we chose the Siemens NX Mechatronic Concept Designer (MCD) environment which enables real-time simulations.

After HVC, the control is applied on the physical device. The controller determines the required angular displacement of particular drives so that the effector achieves the desired position within the manipulator’s coordinate system. The angles are calculated within the TO in PLC and sent to corresponding frequency converters SINAMICS S210 by Siemens. The PLC also performs the safety part of the program over every drive. All safety functions achieve Safety Integrity Level (SIL) 2.

Mechanics of our delta picker robot, designated D4-500-S010 Demo, is manufactured by Codian Robotics. As its working tool, we assume the Bernoulli gripper OGGB by Festo.

### 2.2. Creating Digital Twin in NX MCD

NX MCD is an extension of the CAD software Siemens NX that, among other features, enables to simulate physical behavior of the device. The MCD environment allows to specify which parts will be considered as dynamic objects with physical properties and which will be kept as static objects. This specification plays an essential role in terms of realizing to which extent it is necessary to define the model. As pointed out in the previous section, overdetermination of model would increase computational complexity and hence the length of the operating cycle. In case of devices with fast dynamics, which include also delta manipulators, it is necessary to keep this time as short as possible. That is why we defined only the objects of arms and working tool of the delta robot. For CAD models, the program determines basic parameters such as mass, center of gravity, etc. These can also be set or modified manually, which allows the designers to test various settings. It was also necessary to consider what types of joints are suitable for particular connections. Note that the joints significantly affect the computational complexity.

Signals have been defined as well. They are fed to specific elements of the model by means of UPC UA protocol. There are also functions which enable to calculate some values directly in the software environment, e.g., angular displacement of the motors or position based on data form PLC. In addition, we created signals that are acquired from model and passed to PLC. They substitute the signals from sensors based on events that shall occur for its activation.

### 2.3. Creating Control Program for the Device

In this subsection, we will discuss the advantages of using a single programming environment for designing the entire device. The program for the device will be divided into several parts and for each, these advantages will be pointed out.

#### 2.3.1. Smart Camera

In today’s industry practice, one can encounter a variety of camera types. They are used for object recognition, quality control, as well as position determination—which is also assumed in our case study. Similarly, cameras can be programmed in various languages. The acquired data can be subsequently utilized by other devices, such as in our application. In that case, it is however required that they are all well mastered by a single programmer or that more programmers cooperate. This implies certain mutual dependencies which may lead to undesired delays.

Our camera allows for a simple interface with PLC. Although the program for the camera is created via WBM, it concerns a very intuitive and undemanding programming. Moreover, once the program is created in this environment, it is no longer necessary to use it unless requirements for camera shooting need to be configured, e.g., adding the type of scanned object, etc. All other operations are carried out directly in PLC. After entering the required parameters, these functions also take care of synchronization of the coordinate systems of camera and robot.

**Figure 6:**
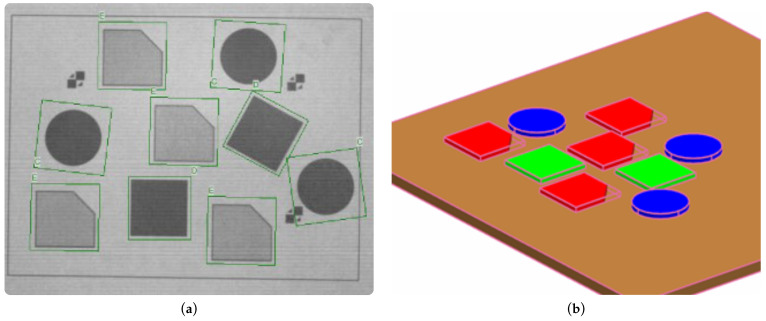
Object recognition using smart camera. (**a**) Detection of parts. (**b**) Visualization of detected parts in NX MCD.

The objects that are to be recognized by the camera are, in reality, printed on a paper that is scanned. This allows to easily alter the scanned scenes. Figure 6a illustrates the object recognition by the camera system. Figure 6b depicts the working surface of the manipulator in the NX MCD environment where a virtual image of the detected objects is created. Therein, the picked-and-placed parts also inherit their physical properties.

#### 2.3.2. Implementation of Kinematic System for Delta Manipulator in PLC

As outlined in Section 2.1, kinematic calculations for the manipulator were carried out within the PLC program. In order to process the TOs, the TIA Portal environment uses motion control (MC) functions. When using an arbitrary TO, organization blocks (OBs) are automatically inserted into the project. These have their own execution level and are called within the MC application cycle. It concerns the MC-SERVO (OB91) and MC-Interpolator (OB92) blocks, which are know-how protected and cannot be unmasked or edited. The calculations are performed in the MC-SERVO OB, which is executed before the MC-Interpolator OB that evaluates MC instructions, generates reference values and monitors functionalities [24].

Among the available TOs within MC, one may choose different object types, while two of them are particularly important for this work. The first object type relates to selecting the function of an axis, which may be set as a speed, positioning or synchronous one. The axis TOs can be used independently to control a single actuator, e.g., an asynchronous motor, or they can be used for a kinematic object, which is the second object type essential for this work. The axis TOs can be simulated directly in the environment, which does not necessitate a connection to the real device.

The kinematic objects, as the name implies, relate to kinematic systems. It is possible to choose from a range of predefined objects and thus to create a custom kinematic system. The predefined systems use preprogrammed kinematic transformations, which only need to be assigned geometric parameters and axis TOs. On the other hand, custom systems require to program kinematic transformations beforehand. A great advantage is the option to employ virtual commissioning directly in the TIA Portal environment. The predefined systems also include the kinematic system of a delta manipulator.

#### 2.3.3. Used Libraries

The Siemens company offers strong software support with its products. Within its Industry Online Support, it provides many libraries for various tasks together with sample application problems. This facilitates the work of programmer and shortens the required time.

To create the program for our device, we used the following libraries and sample applications:

- LKinCtrl [25]—a library that facilitates the work with TOs by providing functions for control of an entire kinematic system and for execution of path motion, as well as sample applications, one of them being a pick-and-place application for a delta manipulator. We used some of its functions and modified them for our purposes.
- Virtual commissioning for kinematics in NX MCD with Software in the Loop [26]—demonstrates how to interface NX MCD with PLC by providing several sample applications, including one for the delta manipulator from which we adopted functions to process data fed to the simulation.
- Transformation of MV440 camera coordinates into robot coordinates [27]—demonstrates how to use the smart camera by describing its integration, the identification procedure and functions for its control. We adopted a function from this application that takes care of the entire communication between PLC, camera and interface for position data from the camera. Other functions are responsible for controlling the camera and conversion between the coordinate systems of the camera and the robot.
- LDrvSafe [28]—a library that provides fail-safe blocks to implement various safety applications. We used it for safety when transitioning to hybrid commissioning.

### 2.4. Communication between Model and Controller Using PLC OPC UA Server

Communication between the PLC and the model was handled by OPC UA, a protocol based on server–client framework. The server provides access to data and functions which are object oriented. The client can access the server via a line with various security levels.

**Figure 7:**
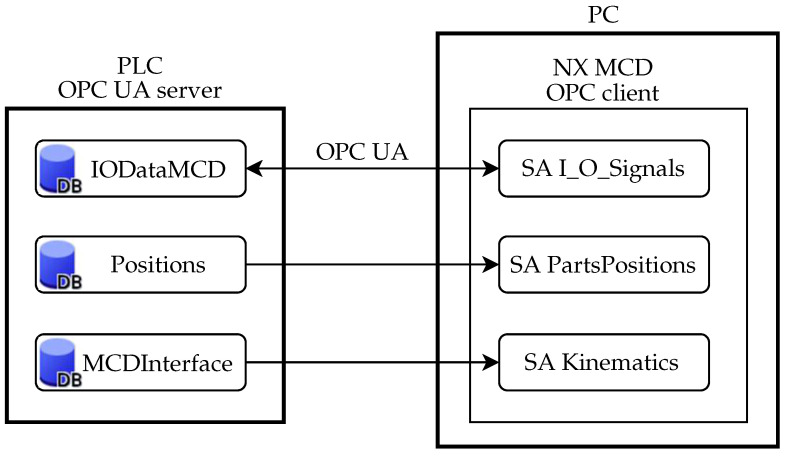
Communication structure between PLC OPC UA server and NX MCD (DB—data block, SA—signal adapter.)

As mentioned in the previous section, the PLC that we employed allows to create an OPC UA server. The initial idea was to use the PLC as the server. Such a server can be assigned a sampling interval. Its minimum value for the given PLC can be set as 100 ms. As shown in Figure 7, PLC contains data blocks which are used by NX MCD to read or log data by means of so-called signal adapters. Particular signals need to be mapped in NX MCD. This is facilitated by the possibility of automatic mapping when signals with the same name in signal adapters and external signals are automatically connected.

When connecting NX MCD to the OPC UA server, it is possible to set an update time, i.e., how often the server data are to be read or logged. As it would not be meaningful to make it shorter than the OPC UA sampling time, they were set as same.

**Figure 8:**
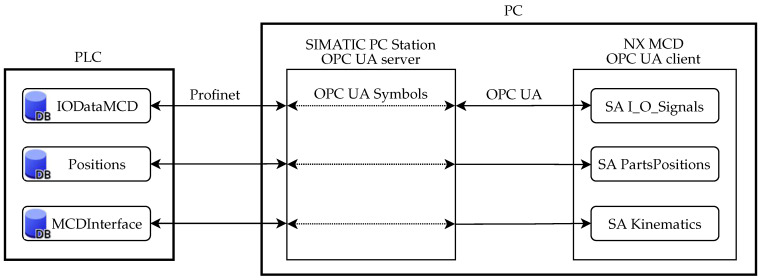
Communication structure between PLC, PC Station and NX MCD.

There was however a fundamental problem with the communication, where the communication speed was not sufficient. We will discuss it in more detail in Section 3.1. It led to using SIMATIC PC Station, which is a software component that manages the SIMATIC software products and interfaces on a PC. It was used to set up an OPC UA NET server that allowed us to achieve a much lower cycle time, which we chose as 20 ms. The impact of cycle time on real-time simulation will be discussed later as well. Note that the change of server also caused the structure of communication between the OPC UA server and the client to change. This structure is shown in Figure 8.

## 3. Results and Discussion

In this section, we present our findings resulting from testing of the DT of our delta manipulator and application of the proposed HVC approach. Within the HVC phase of our case study, we simulate everything except for the camera system and the controller. As outlined in previous sections, all physical devices are simulated either in TIA Portal or NX MCD environment.

**Figure 9:**
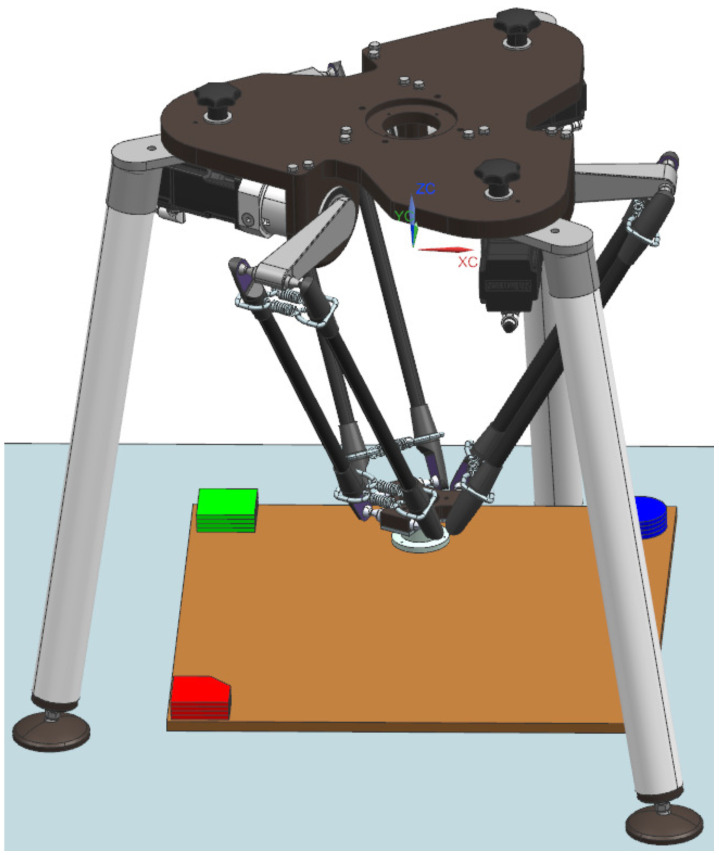
Digital twin of the delta robot manipulator designed in NX MCD environment.

Figure 9 depicts the model of our device designed in NX MCD. In addition to the mechanics itself and the manipulated parts, this DT does not contain a model of the camera system, which from our perspective has no impact on the mechanics and is not simulated in any way.

As we already pointed out, the smart camera that we used allows for easy WBM programming. Importing the library into the control program enabled all the following work in the TIA Portal environment. For using the camera system, it was necessary to synchronize the coordinate systems of robot and camera. Note that the camera’s connection to the system and information obtained from [27] made it easier to tackle this problem. This turned out to be convenient within the next step in which we applied the created program on real mechanics of the delta manipulator. The positions of scanned objects are written to the data blocks which are used for control of the delta robot manipulator. Via the OPC UA server, these data were simultaneously sent to NX MCD, where initial positions of the picked-and-placed parts were set according to scanned images; recall Figure 6.

### 3.1. Hybrid Virtual Commissioning of Robotic Manipulator

**Figure 10:**
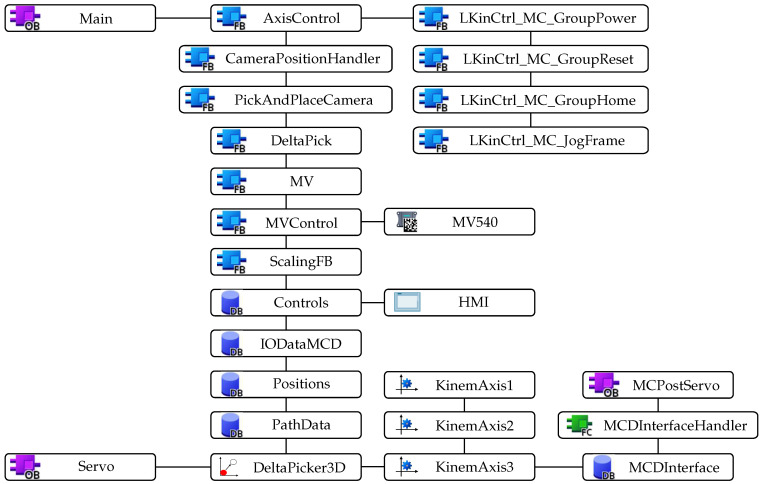
Call structure of the PLC program using HVC. Note: OB—organization block, FB—function block, DB—data block.

The call structure of the PLC program for this testing is depicted in Figure 10. Communication between PLC and NX MCD was taken care of by the PLC OPC UA server as illustrated in Figure 7. To work with the camera, it was necessary to create a program for calibration as well as a program for object recognition. Its objective is to set the position of parts according to the scans obtained from the camera system and, subsequently, to control the manipulator so as to pick and stack the parts in a chosen position. To make the control even more user-friendly, we have also created an HMI which allows us to switch between the programs for the camera as well as to track the evaluated scans.

By testing, we validated the transfer of data for signal adapters of position control, which simulate angular displacement of manipulator’s axes. Next, by comparing simulation of the manipulator in NX MCD with its tracking using Kinematics trace in TIA Portal, we validated synchronization of the coordinate systems.

**Figure 11:**
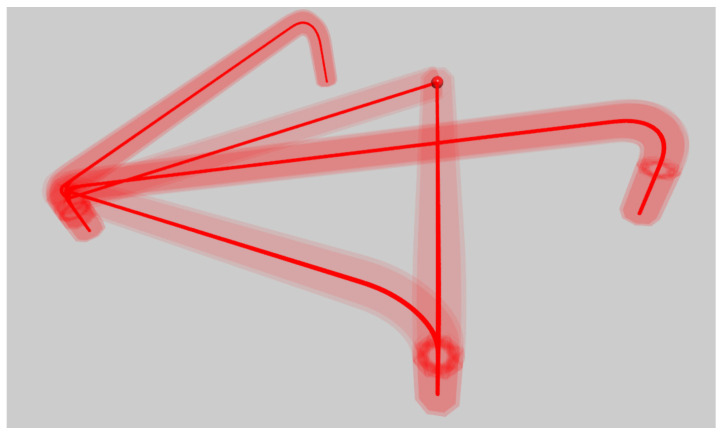
Trace of the tool visualized in TIA Portal.

By means of the DT, we were able to test functionalities, communication between environments and correct command execution in advance. During the testing, we also tracked the trajectory of tool motion in NX MCD and also in Tia Portal; see Figure 11.

**Figure 12:**
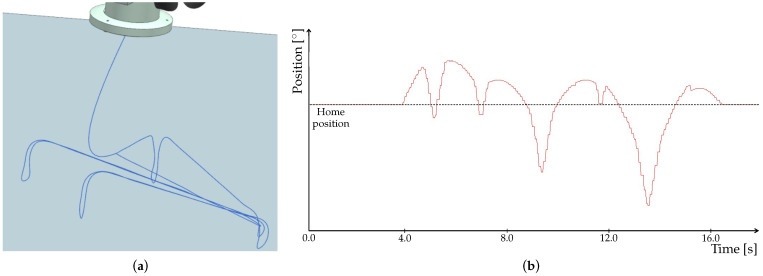
Results from simulation in NX MCD using PLC OPC UA server. (**a**) Trace of the tool. (**b**) Time profile of the signal adapter for axis position control.

The motion of tool in NX MCD however did not follow the programmed trajectory. As shown is Figure 12a, there are loops arising. Since the tool traces in particular environments differed, we included signal adapters for motion control into the tracking in NX MCD. Time profile of the signal is depicted in Figure 12b.

**Figure 13:**
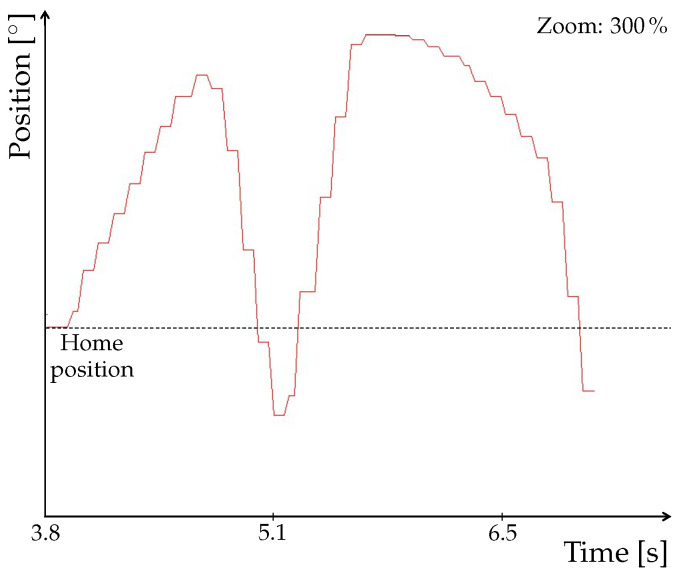
Detail of the signal obtained using PLC OPC UA server.

A detailed look at the signal (Figure 13) clearly shows its step changes which cause the loops in tool trace. In fact, it is due to the communication speed that we mentioned in Section 2.4. This problem thus needs to be solved for verification and validation (V&V) of our model, which we performed by means of animation method. More specifically, we used tracing to observe motion of the tool. For V&V to be acceptable, the tool has to follow the trajectory without any loops. Such a behavior could, in some cases, lead to faulty readings from NX MCD, which would impact the executed program. Therefore, although the program could be correct, deviations in the animation would misrepresent the results and eventually prolong the work.

**Figure 14:**
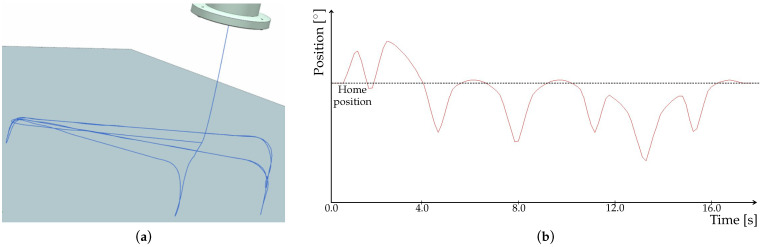
Results from simulation in NX MCD using OPC UA NET server. (**a**) Trace of the tool. (**b**) Time profile of the signal adapter for axis position control.

**Figure 15:**
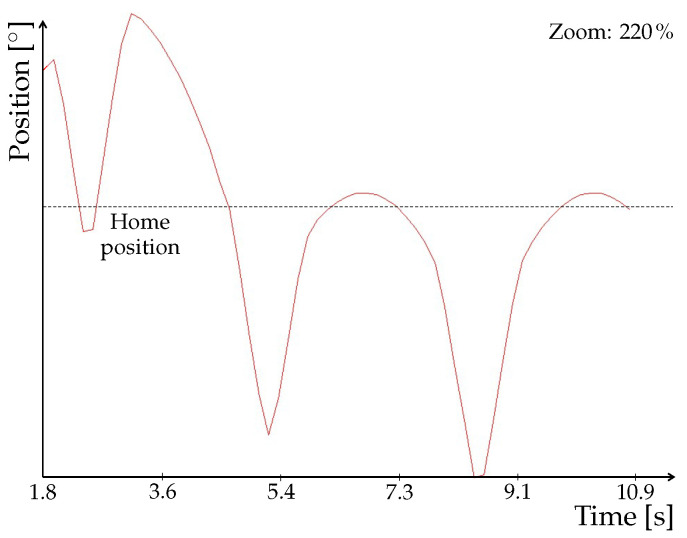
Detail of the signal obtained using OPC UA NET server.

As already mentioned, the OPC PLC UA server was replaced with the faster OPC UA NET server with a cycle time of 20 ms. After this change, the procedure of signal mapping had to be performed again. The update time for NX MCD was also adjusted to match the cycle time of the server. Next, we again observed the tool trace and the signal adapter of one of the position axes. As shown in Figure 14a, the trace no longer contains any apparent loops. Time profile of the signal depicted in Figure 14b is smoother, with no abrupt changes, as shown in detail in Figure 15.

### 3.2. Testing Scenarios

During the tests, we had encountered various errors, from minor to major ones that could damage the device or even endanger safety of persons. One of the revealed errors was an opposite alignment of the coordinate systems used by TIA Portal and NX MCD. We fixed this error by additional recalculation of variables in NX MCD. Another error had been revealed in the code, which allowed us to rectify misalignment of coordinate systems of the camera and the manipulator. The errors that could cause damage to the real device included one that could make the arms overturn and hence collide with the base and axes of the manipulator. At the same time, it was possible to test how lighting affects the camera resolution and hence the scanned parts. Already in the design phase, this helped us to better understand what the optimal conditions for accurate object recognition are.

Hybrid virtual commissioning may however entail a disadvantage in combination with available real-time simulation capabilities. In the current implementation, real-time simulation for systems with fast dynamics, such as the delta robot, is due to limited computational power not feasible for a full speed of motion attainable by the device.

### 3.3. Partial Application on Real Manipulator

**Figure 16:**
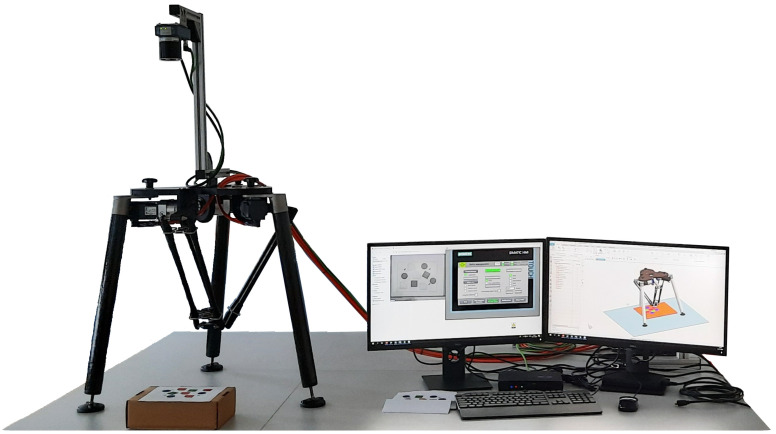
Real, partially implemented delta robot manipulator and its digital twin implemented in NX MCD.

Finally, we implemented the developed and by-HVC-validated program to control the physical device. When transitioning to the real robot manipulator, safety functions were added to the project and virtual axes were replaced with frequency converters of the SIMATIC S210 series. Since we had not had the gripper components at our disposal, its function remained in NX MCD where signals indicating gripping were obtained from. Figure 16 depicts the real, partially implemented delta robot manipulator together with its digital twin implemented in NX MCD and an HMI for controlling the manipulator.

After commissioning of the device, the DT can be reused, for example, when testing a new type of work tool. This will shorten the time required for changing the work tool of the real device. It can also be used for integration into a production line, where it will collaborate with other devices on performing given tasks, or for testing the feasibility of new trajectories when modifying the control program.

## 4. Conclusions

Digital twins offer a great potential by predicting future behavior of manufacturing systems and processes instead of analyzing the past. When combined with appropriate equipment, it is possible to obtain more from the DT than what was invested in it. It is therefore no surprise that there is an ongoing effort to make processes and their implementation more efficient. It is the implementation of solutions where one may observe a diversity of environments and control approaches which allow to achieve the desired result. This diversity often introduces additional requirements into the implementation of a solution, which can lead to an increase in cost, required time or personnel. Hence, there is also an effort to make the time from designing up to commissioning as short as possible. This can be achieved already in the design stage by means of testing using various models—mathematical, statistical or animative ones. These models are subsequently subject to the aforementioned approaches of VC or HC.

Our proposed approach of HVC represents a concept that combines properties of VC and HC. It makes sense to use it if the cost of simulating a device was higher or the work required to model a certain subsystem was more demanding than direct implementation of a particular device into the model. The HVC concept can be conveniently used as a tool in the training and teaching process. Thanks to virtualization and simulation of processes, there is no risk of damaging equipment or causing injuries.

The HVC concept has been demonstrated in a case study featuring a robotic manipulator with machine vision and a single PLC in a pick-and-place application. Its DT created in NX MCD allowed to accelerate the commissioning. At the same time, during verification and validation of the control program, using an animation method, we were able to reveal certain errors before implementing the program on a real device. Besides the concept itself, we also used the case study to demonstrate the benefits of using fewer software environments. In particular, we used three software environments, one of them via WBN. This implies fewer programmers, although the requirement on programmer’s knowledge is higher.

Our further research will focus on the possibility of using digital twin to determine suitability of a device for improving a given process.

## Data Availability

The data presented in this study are available on request from the corresponding author. The data are not publicly available due to privacy.
